# Spatio-temporal and dynamic regulation of neurofascin alternative splicing in mouse cerebellar neurons

**DOI:** 10.1038/s41598-017-11319-5

**Published:** 2017-09-12

**Authors:** Satoko Suzuki, Noriko Ayukawa, Chisa Okada, Masami Tanaka, Susumu Takekoshi, Yoko Iijima, Takatoshi Iijima

**Affiliations:** 1 Tokai University Institute of Innovative Science and Technology, 143 Shimokasuya, Isehara City, Kanagawa 259-1193, Japan; and 411 Kitakaname, Hiratsuka City, Kanagawa 259-1292 Japan; 2 Support Center for Medical Research and Education, Tokai University, 143 Shimokasuya, Isehara City, Kanagawa 259-1193 Japan; 3 Department of Cell Biology, Division of Host Defense Mechanism, School of Medicine, Tokai University, 143 Shimokasuya, Isehara City, Kanagawa 259-1193 Japan

## Abstract

Alternative splicing is crucial for molecular diversification, which greatly contributes to the complexity and specificity of neural functions in the central nervous system (CNS). Neurofascin (NF) is a polymorphic cell surface protein that has a number of splicing isoforms. As the alternative splicing of the neurofascin gene (*Nfasc*) is developmentally regulated, NF isoforms have distinct functions in immature and mature brains. However, the molecular mechanisms underlying the alternative splicing of *Nfasc* in neurons are not yet understood. Here, we demonstrate that, alongside developmental regulation, *Nfasc* alternative splicing is spatially controlled in the mouse brain. We then identified distinct *Nfasc* splicing patterns at the cell-type level in the cerebellum, with *Nfasc186* being expressed in Purkinje cells and absent from granule cells (GCs). Furthermore, we show that high K^+^-induced depolarization triggers a shift in splicing from *Nfasc140* to *Nfasc186* in cerebellar GCs. Finally, we identified a neural RNA-binding protein, Rbfox, as a key player in neural NF isoform selection, specifically controlling splicing at exons 26−29. Together, our results show that *Nfasc* alternative splicing is spatio-temporally and dynamically regulated in cerebellar neurons. Our findings provide profound insight into the mechanisms underlying the functional diversity of neuronal cell-adhesive proteins in the mammalian CNS.

## Introduction

Alternative pre-mRNA splicing is a fundamental mechanism that generates molecular diversity from a single gene, and is therefore thought to be essential for biological complexity and diversity. The regulation of this splicing is highly dynamic and complex in the vertebrate central nervous system (CNS)^1, 2^. Alternative splicing decisions are known to be dynamically switched during neural development^3^, and show distinct patterns in a neuronal tissue- or cell type-specific manner^4^. Furthermore, neuronal activity modulates alternative splicing of neural genes via Ca^2+^-dependent signalling pathways^5^. Thus, neuronal alternative splicing is controlled in a spatio-temporal manner, which likely contributes to the complexity and specificity of neural circuits^4, 6^.

Neurofascin (NF) is a polymorphic cell-surface protein that belongs to the L1 subgroup of the immunoglobulin superfamily^7^. Alternative pre-mRNA splicing of the neurofascin (*Nfasc*) gene is developmentally regulated, which results in 50 variant isoforms expressed at different developmental stages^8^. In the CNS and peripheral nervous system, NF is involved in a variety of processes, such as cell adhesion, cell migration, and neurite outgrowth, during development, and is required for action potential propagation and myelination in adults^9^. Thus, the alternative splicing of certain cell-adhesion molecules is thought to underlie the specificity of neuron–neuron and neuron–glia contacts during development, playing a major role in the organization of neural circuits.

Alternative splicing at segments on the proximal ectodomain is particularly crucial for generating the four major NF isoforms, namely NF186, NF180, NF155, and NF140 (NF166 in chicken) in the mammalian CNS^7^. NF186, NF180, and NF140 are neural isoforms, whereas NF155 is a glial isoform. NF consists of a set of six Ig-like domains that are common to all its isoforms and up to five variable FNIII-like domains. The four NF isoforms differ in their combination of FNIII-like domains, as well as in the presence of a PAT domain. Thus, alternatively spliced events provide most of the structural diversity of the NF ectodomain.

The three neural NFs show distinct functions in developing and adult brains^7^. For example, the major neural isoform, NF186, predominantly confers stabilization to axon initial segments (AIS) and nodes of Ranvier in adults. The other neural isoforms, NF140 and NF180, are embryonic protein variants that regulate neurite outgrowth. In accordance with their distinct function, the embryonic isoforms are largely converted to the adult NF186 isoform during neural development and differentiation. This process occurs through the inclusion of four tandem exons (i.e., exons 26, 27, 28, and 29; ex26-29) which encode the fifth FNIII domain and the PAT domain^8^. Thus, alternatively spliced NF isoforms serve as a developmental switch, imparting neural functions that are distinct between developing and adult brains^7^. However, the detailed molecular mechanisms underlying the alternative splicing of *Nfasc* are unknown.

This study shows spatially-dependent and dynamic alternative splicing of neural *Nfasc* in mammals; this regulation is dependent on brain region, neuronal cell type, and activity. Such splicing is likely crucial for creating the morphological and functional diversity of the mammalian CNS.

## Results

### Alternative splicing of Nfasc shows spatial patterning in adult brains

**Figure 1:**
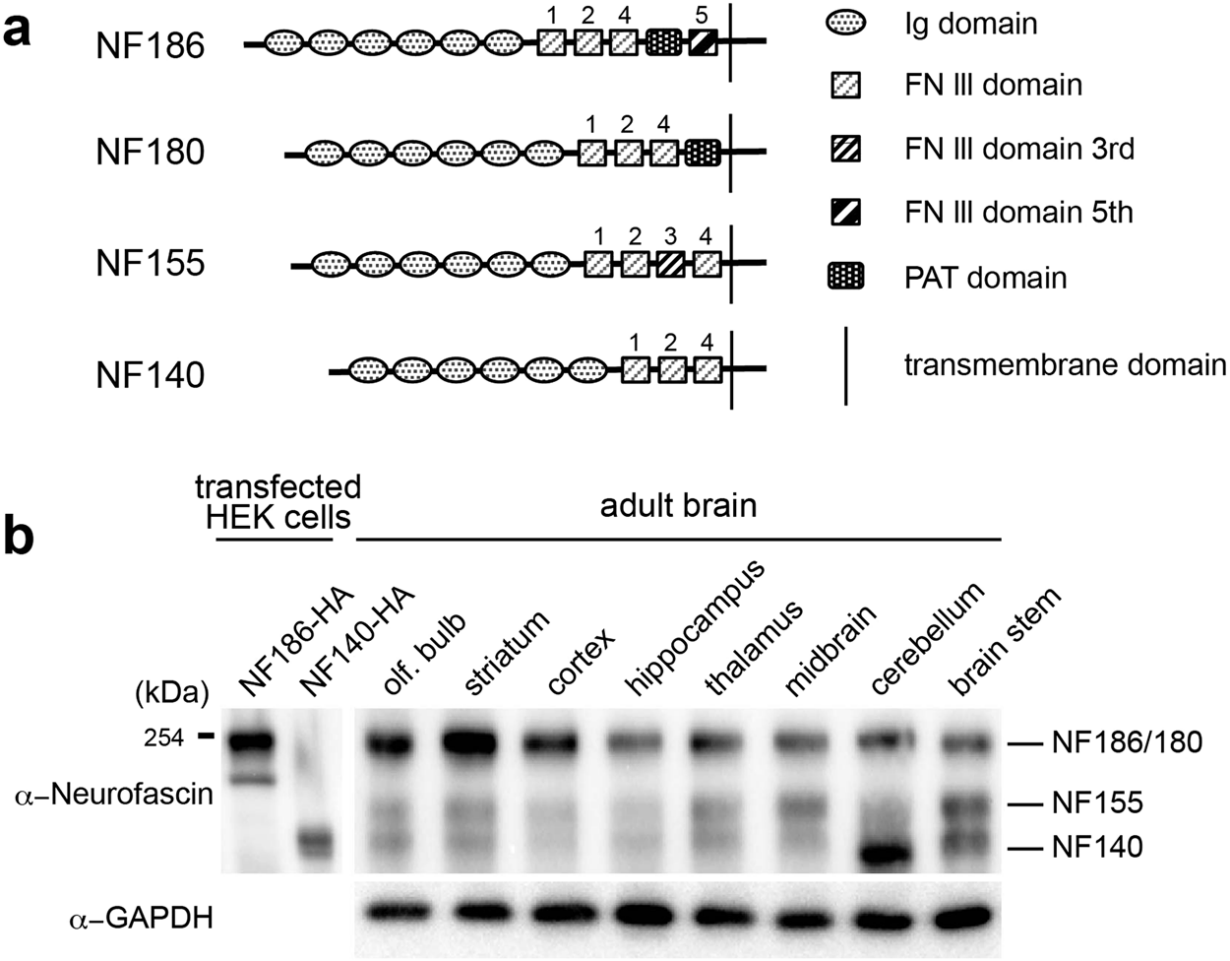
Brain area survey for neurofascin (NF) protein isoforms. (**a**) Schematic diagram outlining four NF protein isoforms. NF proteins consist of six Ig-like domains with different combinations of FNIII-like domains in the ectodomain. (**b**) Expression pattern of NF isoforms in various brain regions as assayed by western blot with the pan-NF antibody. HA-tagged recombinant NF186 and NF140 expressed in HEK293T cells were simultaneously loaded as a control for molecular size.

Alternative splicing of *Nfasc* generates four protein isoforms (NF186, NF180, NF155, and NF140) in the CNS (Fig. 1a). The third FNIII domain (FN3) is unique to glial isoform NF155. However, the PAT domain is included in the neural isoforms NF186 and NF180, with NF186 further including a fifth FNIII domain (FN5). Another neural isoform, NF140, does not contain any of these domains. NF140 is an embryonic isoform, originally identified as NF166 in the chick dorsal root ganglia (DRG)^8^. The NF180 variant is also expressed in the rat brain during early development^10^. In contrast, NF186 is a major isoform expressed in adult brains. Interestingly, both embryonic isoforms shift to the adult isoform, NF186, during development. To verify that NF186 is ubiquitously dominant in adult brains, we performed immunoblotting analysis with an anti-pan-neurofascin antibody in various adult brain tissues; the antibody detects several protein bands around 150–200 kDa (Fig. 1b). Of the protein variants we investigated, the one with the highest molecular weight (i.e., NF186/NF180) was prominent (Fig. 1b). However, we noticed that hindbrain areas, such as the cerebellum and brainstem, displayed higher levels of the lower molecular weight protein (i.e., NF140) (Fig. 1b), which has been reported to be expressed in developing brains^11^. These results revealed a regional difference in the ratio of neural NF isoforms in adult brains.

**Figure 2:**
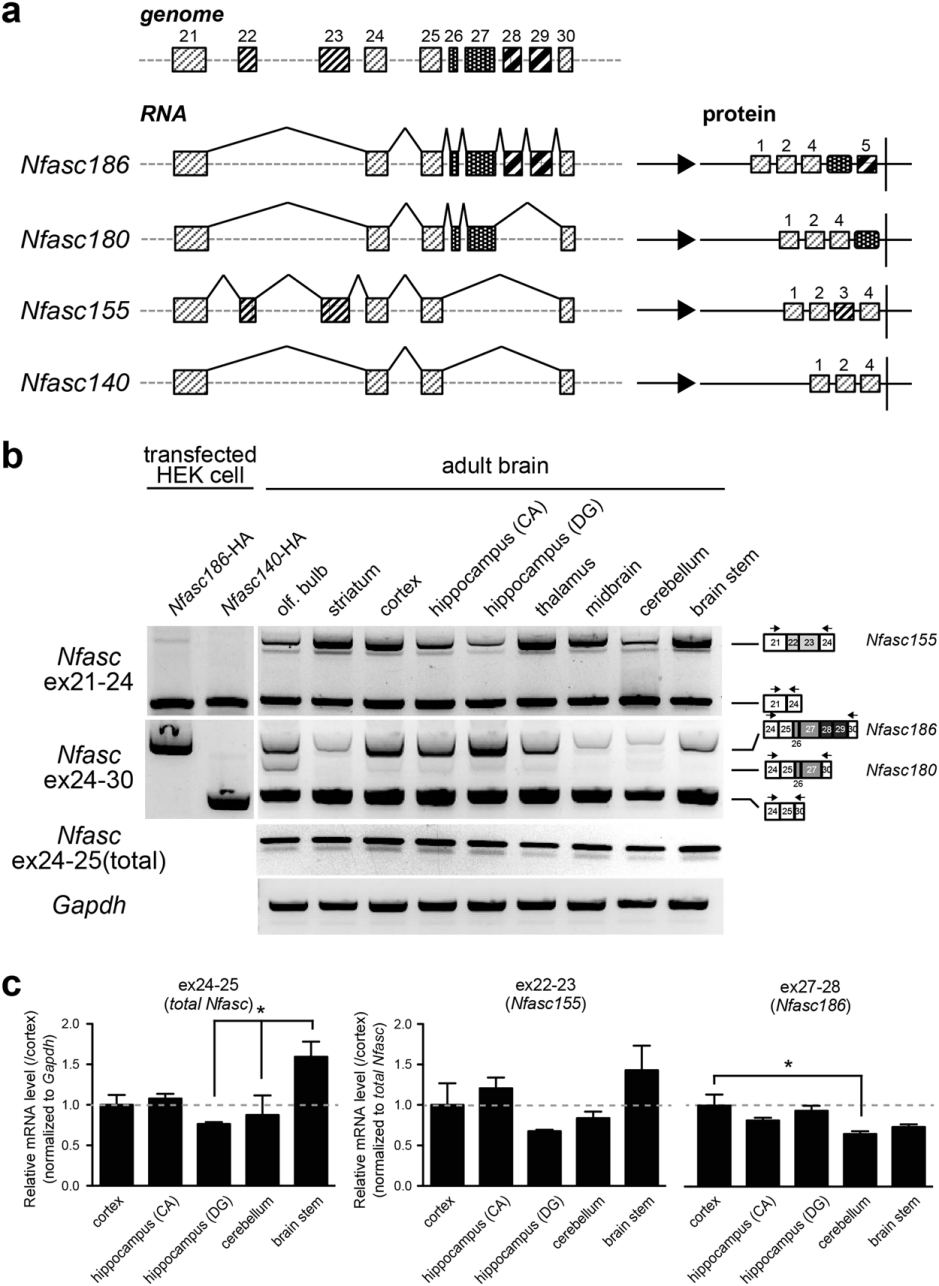
Distinct neuronal neurofascin gene (Nfasc) splicing across the adult brain. (**a**) Schematic diagram outlining partial genomic organization of *Nfasc* at ex21-30 and the alternative splicing patterns that generate four protein splice isoforms. Exon positions of each alternatively spliced segment (ex22-23, ex26-27, and ex28-29) are indicated by different boxes. (**b**) Detection of alternative splicing choices at ex26-29 (neuron-specific splices) by semi-quantitative RT-PCR. Primer sets used for the analysis were designed on the neighbouring constitutive exon sequence. Different splicing patterns at ex26-29 among brain areas was largely consistent with the pattern of protein isoforms in data shown in Fig. 1 (n = 3 animals). (**c**) Quantification of total *Nfasc* (ex24-25), *Nfasc155* (ex22-23), and *Nfasc186* (ex27-28) levels by RT-qPCR. Total *Nfasc*: F (4,14) = 3.919 (P < 0.05); *Nfasc155*: F (4,14) = 2.188 (P = 0.15); *Nfasc186*: F (4,14) = 4.581 (P < 0.05), one-way ANOVA. Asterisks show the significant differences when compared between all the tissues with post-hoc test (Bonferroni’s multiple comparison test) (*n* = 3 animals).

Expression of the three neural NF isoforms is mainly determined by alternative exclusion or insertion of the four tandem exons, ex26-29, which encode the FN5 and PAT domains (Fig. 2a). For example, *Nfasc186* includes all the tandem exons, while *Nfasc180* selectively includes two exons: exons 26 and 27. In contrast, *Nfasc140* does not include any of these exons.

To examine differential expression of neural NF isoforms between adult brain regions further at the “transcript level,” we analysed alternative splicing at ex26-29 by semi-quantitative reverse transcription polymerase chain reaction (RT-PCR). To this end, we designed primer sets to recognize neighbouring constitutive exons (Table 1) and, as expected, we observed three PCR products corresponding to *Nfasc186*, *Nfasc180*, and *Nfasc140/155* (Fig. 2b). A sequence analysis confirmed that these PCR products were derived from the transcripts of *Nfasc* splicing variants (data not shown). Moreover, the expression analyses derived by RT-PCR revealed that the splicing pattern of ex26-29 varied between brain areas, implying that *Nfasc186* transcripts, which include ex26-29, were more abundantly expressed in forebrain areas (Fig. 2c, right graph). Furthermore, with the exception of the olfactory bulb, *Nfasc180* transcripts, which include exons 26 and 27, were nearly undetectable. This finding suggests that the higher molecular weight bands observed in Fig. 1 could not contain significant amounts of NF180, except for some brain areas. In addition, we evaluated splicing at exons 22 and 23 (ex22-23), as this pattern could lead to expression of glial *Nfasc155*. Differences in ex22-23 splicing among adult brain areas were not as evident as those of ex26-29 (Fig. 2b), suggesting that alternative splicing at ex26-29—a segment critical for expression of the neuronal *Nfasc* isoform—is spatially regulated in adult brains.

**Table 1:** Oligonucleotide sequences of PCR primer sets.

Primer (Forward) (Reverse)	Sequence (5′–3′)
*Nfasc* ex24-F	5′-ATA CAT CCT CAG ATA CGT GC-3′
*Nfasc* ex30-R	5′-TGC CTG GTT ATT GGT GTA AG-3′
*Nfasc* ex21-F	5′-CAC CAT CAT TGG GTA CTC C-3′
*Nfasc* ex24-R	5′-AGG GCA CGT ATC TGA GGA TG-3′
*Bdnf*-F	5′-GGA CAT GTC TGG CGG GAC GGT C-3′
*Bdnf*-R	5′-CTA TCT TCC CCT TTT AAT GGT CAG-3′
*Gapdh*-F	5′-TGT TGC CAT CAA TGA CC-3′
*Gapdh*-R	5′-TCT CAT GGT TCA CAC CCA-3′
*Nfasc* ex22-F(qPCR)	5′-CTG AAC AGC ACA GCC ATC AG-3′
*Nfasc* ex23-R(qPCR)	5′-ACA CCC ACA GGT TCT TCA GC-3′
*Nfasc* ex24-F(qPCR)	5′-ACCTGGAGACCATCAACCTG-3′
*Nfasc* ex25-R(qPCR)	5′-TTT TCC ACC ATC TGC TTT CC-3′
*Nfasc* ex27-F1(qPCR)	5′-AAG CCA CAA CAG TTC CCA TC-3′
*Nfasc* ex27-R(qPCR)	5′-CTC TCC GTA GTG GTG GTG GT-3′
*Nfasc* ex27-F2(qPCR)	5′-AGA GCC CTC CCA CTA CCA CT-3′
*Nfasc* ex28-R(qPCR)	5′-GGT GAT GTT GGC CCA TTT AC-3′
*Rbfox1*-F(qPCR)	5′-TAA CTT TCG AAA ATA GTG CG-3′
*Rbfox1*-R(qPCR)	5′-GGG TTG ACA GTC TTC TTA TT-3′
*Rbfox3*-F(qPCR)	5′-GCA CAG ACT CAT CCT GAG CA-3′
*Rbfox3*-R(qPCR)	5′-GGT GGA GTT GCT GGT TGT CT-3′
*Gapdh*-F(qPCR)	5′-TGT TCC AGT ATG ACT CCA CTC ACG-3′
*Gapdh*-R(qPCR)	5′-AGT AGA CTC CAC GAC ATA CTC AGC-3′

However, in contrast to the protein analysis results shown in Fig. 1, *Nfasc140/155* transcripts, which lack ex26-29, were strongly amplified from all brain areas in semi-quantitative PCR. The discrepancy might be due to a large difference in PCR amplification between longer and shorter variants, because the length of the products differed by more than 500 bp. Therefore, we conducted quantitative RT-PCR (qRT-PCR) to quantify the transcript levels of *Nfasc186* and *Nfasc155* more exactly between three forebrain and two hindbrain areas. There was a significant variance in the total *Nfasc* levels (ex24-25) between brain areas (Fig. 2c left). However, the relative transcript level of ex27-28, selectively included in *Nfasc186* transcript, (normalized to total *Nfasc*) was relatively lower in the two hindbrain areas than in the three forebrain areas (Fig. 2c right), similar to the pattern obtained by semiquantitative PCR in Fig. 2b. While there was no significant difference in *Nfasc155* (ex22-23) between the brain areas (Fig. 2c middle), *Nfasc186* levels in the cerebellum was significantly lower than that in the cortex, by approximately 35% (Fig. 2c right), confirming the regional difference in neuronal *Nfasc* isoforms.

### Alternative splicing of Nfasc is cell type-specific in cerebellar neurons

**Figure 3:**
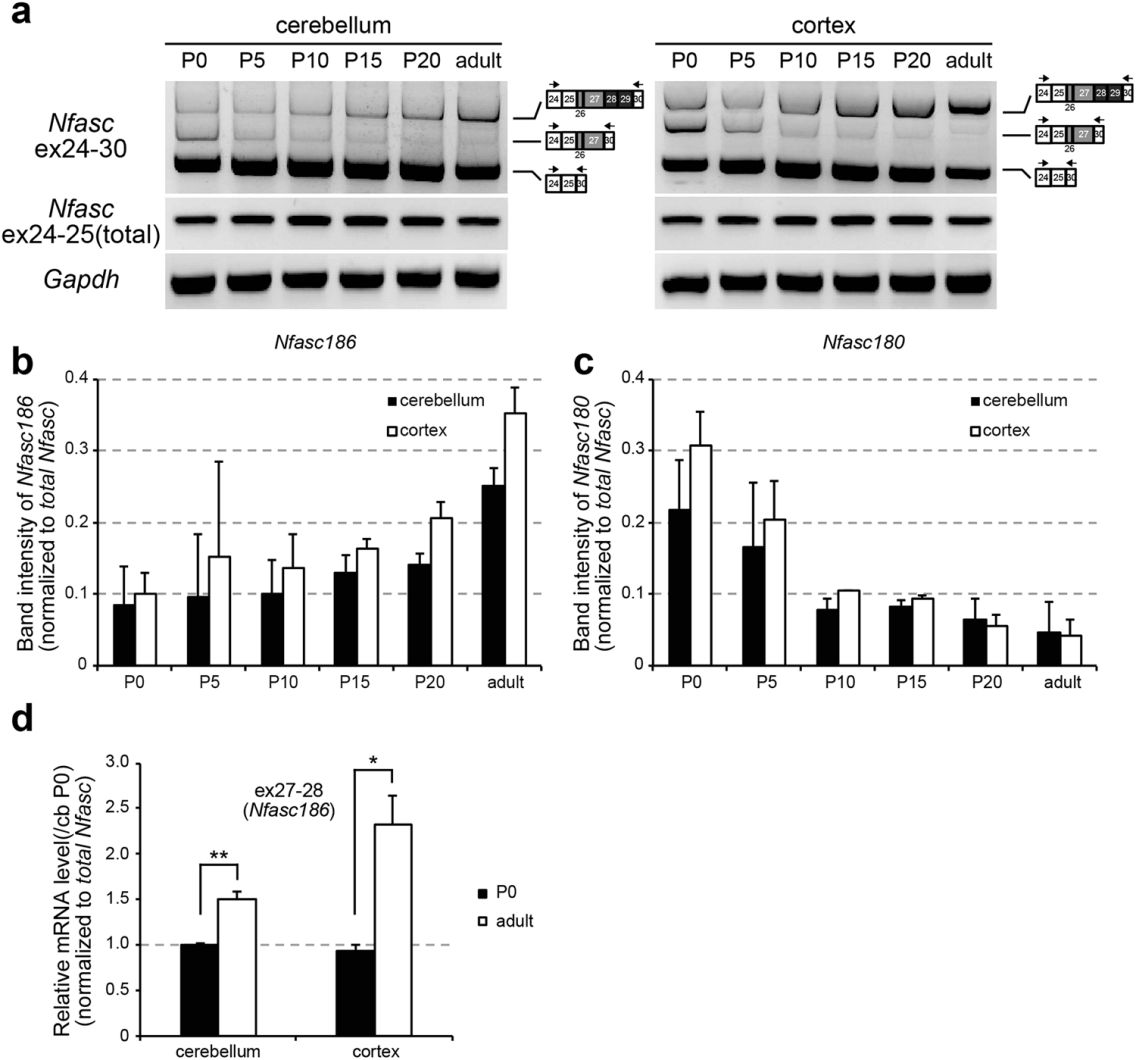
Developmental change and alternative splicing of neuronal Nfasc in the adult cerebellum. (**a**) Developmental profile of ex26-29 inclusion in the cerebellum and cortex. The amount of each neuronal *Nfasc* isoform were changed dynamically during development. (**b,c**) Quantification of *Nfasc180* and *Nfasc186* levels by semi quantitative PCR to produce the results shown in (**a**). (**b**) *Nfasc186*. (**c**) *Nfasc180*. Band intensity of each *Nfasc* isoform was normalized to that of *total Nfasc* (ex24-25). (**d**) Quantification of ex27-28 inclusion (*Nfasc186*) at postnatal day 0 (P0) and adulthood (2–3-months-old) in the cortex and cerebellum by qRT-PCR. (*n* = 3 animals) Values for cerebellum at P0 were set to 1.0.

To investigate the mechanisms underlying the spatial difference in neural NF isoform expression among brain areas, we focused on the large disparity in NF variants between the cortex and cerebellum. Whereas the adult cortex predominantly and strongly expressed NF186, the adult cerebellum inversely expressed the embryonic isoform NF140 (Fig. 1). Therefore, we compared *Nfasc* splice selection during cerebellar and cortical development. As expected, *Nfasc186* gradually increased (Fig. 3a,b), whereas *Nfasc180* reciprocally decreased (Fig. 3a,c) during cortical and cerebellar development to different extent, respectively. The qRT-PCR confirmed the significant differences in inclusion of ex27-28 between postnatal day 0 and adulthood (2-3 months) (Fig. 3d). These data indicate that the amounts of *Nfasc* isoforms changed distinctly during cortical and cerebellar development, through inclusion of ex26-29, but the extent of the developmental change in the cerebellum was less than that in the cortex (Fig. 3d).

**Figure 4:**
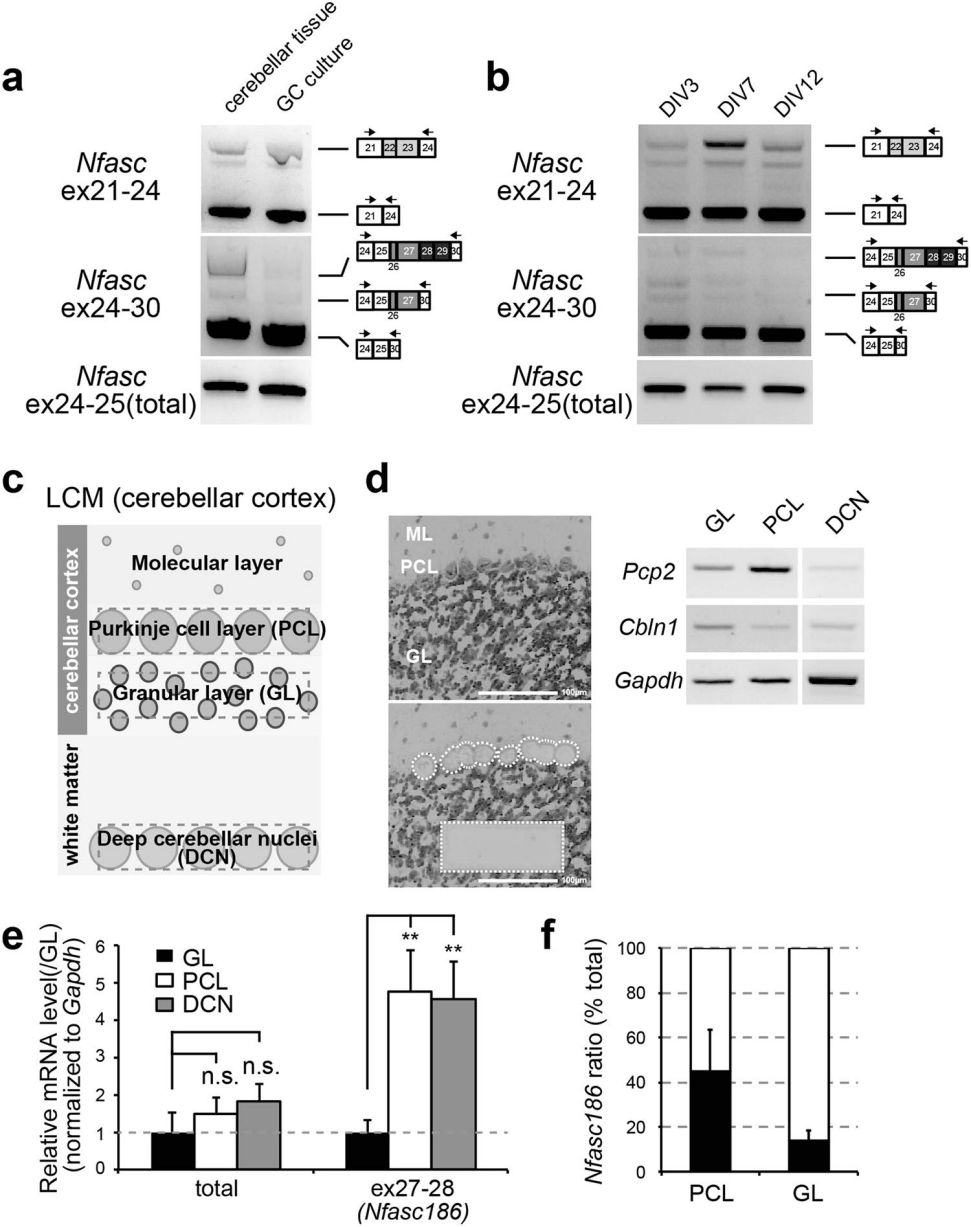
Cell type–specific alternative splicing of Nfasc in the adult cerebellum. (**a**) Comparison of the inclusion patterns at ex22-23 and ex26-29 between *Nfasc* in whole cerebellar tissue and in granule-cell (GC) cultures. Inclusion of ex26-29 was remarkably less in GC cultures. (**b**) Inclusion patterns at *Nfasc* ex22-23 and ex26-29 between time points in developing GC cultures. Inclusion of ex26-29 did not occur developmentally in GC cultures. (**c**) Illustration of separation and cell isolation by laser-capture microdissection (LCM). The Purkinje cell (PC) layer (PCL), granule layer (GL), and deep cerebellar nuclei (DCN) were isolated from single cerebellar slices. (**d**) Representative images of LCM isolates in the cerebellar cortex and verification by standard PCR using primer sets for cell type-specific markers; *Pcp2* (for PCs) and *Cbln1* (for GCs). Scale bar = 100 μm. (**e**) RT-qPCR analysis using LCM-isolated cerebellar tissue. *Nfasc186* containing ex27-28 was 4–5 fold higher in the PCL and DCN than in the GL (*n* = 4 animals). Values for GL were set to 1.0. Differences were compared to the values for GL. (**f**) A ratio of *Nfasc186* in the PCL and GL by qRT-PCR analysis (*n* = 4 animals). Approximate percentage of *Nfasc186* isoform was largely estimated from CT value of ex27-28 directly compared to that of total *Nfasc* (ex24-25) at the same threshold set for CT value. Mean CT values for total *Nfasc* were set to 100%.

We then analysed alternative splicing choices for *Nfasc* at the neuronal cell-type level in the adult cerebellum. One major type of neuron in the cerebellum is the granule cell (GC); thus, we compared alternative *Nfasc* splicing at ex26-29 between cerebellar tissue and GC cultures using standard RT-PCR. Interestingly, while *Nfasc186* transcripts were not detectable in GC cultures, a small amount was identified in whole cerebellar tissue (Fig. 4a), suggesting that *Nfasc186* is remarkably less from GCs. We further investigated the inclusion of ex26-29 in immature GC cultures (at 3 days in culture [DIV] and 7 DIV) and confirmed that ex26-29 was not largely included in *Nfasc* at any time in cerebellar GC development (Fig. 4b).

The latter finding implies that the small amount of *Nfasc186* observed in the cerebellum was derived from alternative cerebellar neurons. Previous reports have shown that NF186 is expressed in Purkinje cells in the adult cerebellum, and the variant plays an important role in AIS organization and function^12, 13^. Therefore, we separated three cerebellar areas (granule layer [GL], Purkinje cell layer [PCL], and deep cerebellar nuclei [DCN]) by laser capture microdissection (LCM) (Fig. 4c,d), and compared ex27-28 inclusions among them. qRT-PCR analyses showed that ex27-28 inclusion levels were four- to five-fold higher in the PCL and DCN than in the GL (Fig. 4e). The qRT-PCR study also estimated that at least 40–50% of *Nfasc* transcripts in Purkinje cells were of the *Nfasc186* isoform, whereas *Nfasc186* accounted for less than 15% of *Nfasc* transcripts in cerebellar GCs (Fig. 4f). These findings demonstrate cell type-specific regulation of neuronal NF isoforms in the adult cerebellum.

### High K^+^-induced depolarization triggers a shift in alternative splicing of Nfasc in cerebellar GCs

**Figure 5:**
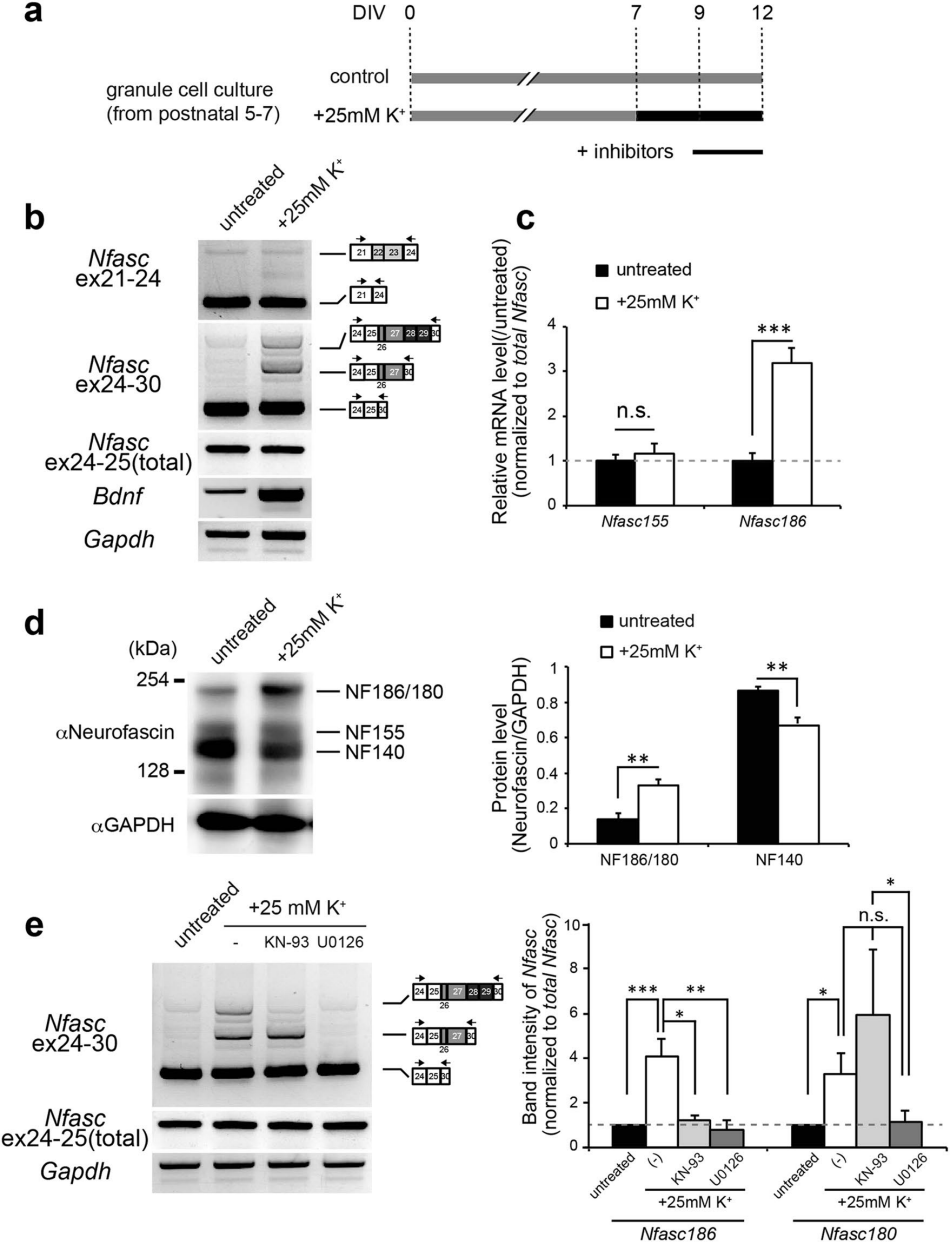
Depolarization-dependent alternative splicing of neuronal *Nfasc* in cerebellar GCs. (**a**) Schematic diagram of the *in vitro* pharmacological experiment. Cerebellar GCs were maintained in normal K^+^ media (5 mM, control; grey bar) or high-K^+^ media (final 30 mM, intervention; black bar) from 7 days in culture (DIV) through 12 DIV. The pharmacological agents were applied for 3 days before harvest (under black line). (**b**) Representative images of switch in the splicing of *Nfasc* caused by high K^+^-induced depolarization. Depolarization strongly induced inclusion of ex26-29 in cerebellar GCs. Brain-derived neurotrophic factor (*Bdnf*) expression was monitored to confirm immediate-early gene induction. (**c**) RT-qPCR analysis of the depolarized GC culture. Ex27-28, included in *Nfasc186*, was increased as a result of high-K^+^ stimulation. (*n* = 6 cultures) Values for the untreated culture were set to 1.0. (**d**) Western blot analysis of total cell lysates from untreated control and depolarized cultures with anti-neurofascin antibody. Shown is a shift in neuronal NF isoform in cerebellar GC culture due to high K^+^-induced depolarization. Intensities of protein bands corresponding to NF186 and NF140 were normalized to that of GAPDH. (*n* = 3 cultures). (**e**) Pharmacological experiments in depolarized GC cultures. KN-93 (CaMK selective inhibitor, 10 µM), and U0126 (ERK inhibitor, 5 µM) were used to block Ca^2+^-dependent signalling. Values for untreated GCs were arbitrarily defined as 1.0. (*n* > 4 cultures). *Nfasc186*: F (3,24) = 8.374 (P < 0.0001); *Nfasc180*: F (3,36) = 8.423 (P < 0.0001), one-way ANOVA. Differences were compared to the untreated group using the post-hoc test (Dunnett’s test), after one-way ANOVA.

Although *Nfasc* splicing in cerebellar GCs was not changed during development, we speculated whether *Nfasc* splicing was regulated via additional mechanisms in cerebellar GCs. Increasing evidence suggests that a significant number of neural proteins are subject to alternative splicing in response to neuronal activity^5^. We have previously shown that depolarization/neuronal activity dynamically modulates alternative splicing events in cerebellar GCs^14^. Therefore, we next tested the inducible change in *Nfasc* splicing in cerebellar GCs, by applying chronic high K^+^ treatment as shown in Fig. 5a. Interestingly, we found that high-K^+^ stimulation over a period of several days induced inclusion of *Nfasc* ex26-29 in transcripts, leading to an increase in *Nfasc186/180* in cultured GCs (Fig. 5b). Bands of *Nfasc186* became detectable after incubation for 1 day or more (Fig. S1). Thus, inclusion of *Nfasc* ex26-29 was dependent on the duration of high K^+^ treatment. Analysis by qRT-PCR revealed that inclusion of *Nfasc* ex26-29 significantly increased, by approximately three-fold, in the 5-day depolarization protocol, as shown in Fig. 5a (Fig. 5c). In contrast, the splicing of glial *Nfasc155* (which includes ex22-23) was not changed by the high K^+^ stimulation (Figs 5c, S1). Importantly, immunoblot analysis with pan-NF antibody showed that high-K^+^ stimulation significantly increased NF186/180 and reciprocally reduced NF140 (Fig. 5d), confirming the depolarization-dependent change in *Nfasc* splicing at the protein level.

We next determined which cellular signalling pathway was critical for *Nfasc* splicing at ex26-29. In pharmacological experiments with two kinase inhibitors, the ERK inhibitor U0126 markedly decreased the depolarization-dependent shift to *Nfasc186*, indicating a role for the ERK/MAPK pathway in mediating the high K^+^-induced insertion of ex26-29 (Fig. 5e). Moreover, the CaMK inhibitor, KN-93, selectively inhibited *Nfasc186* expression, indicating that the CaMK pathway selectively mediates the inclusion of ex28-29, but not that of ex26-27 (Fig. 5e). Together, these data suggest that *Nfasc* splicing at ex26-29 is regulated by depolarization/neuronal activity synergistically via both CaMK and ERK/MAPK signalling.

### The Rbfox family is responsible for neural isoform selection through inclusion of Nfasc ex26-29

**Figure 6:**
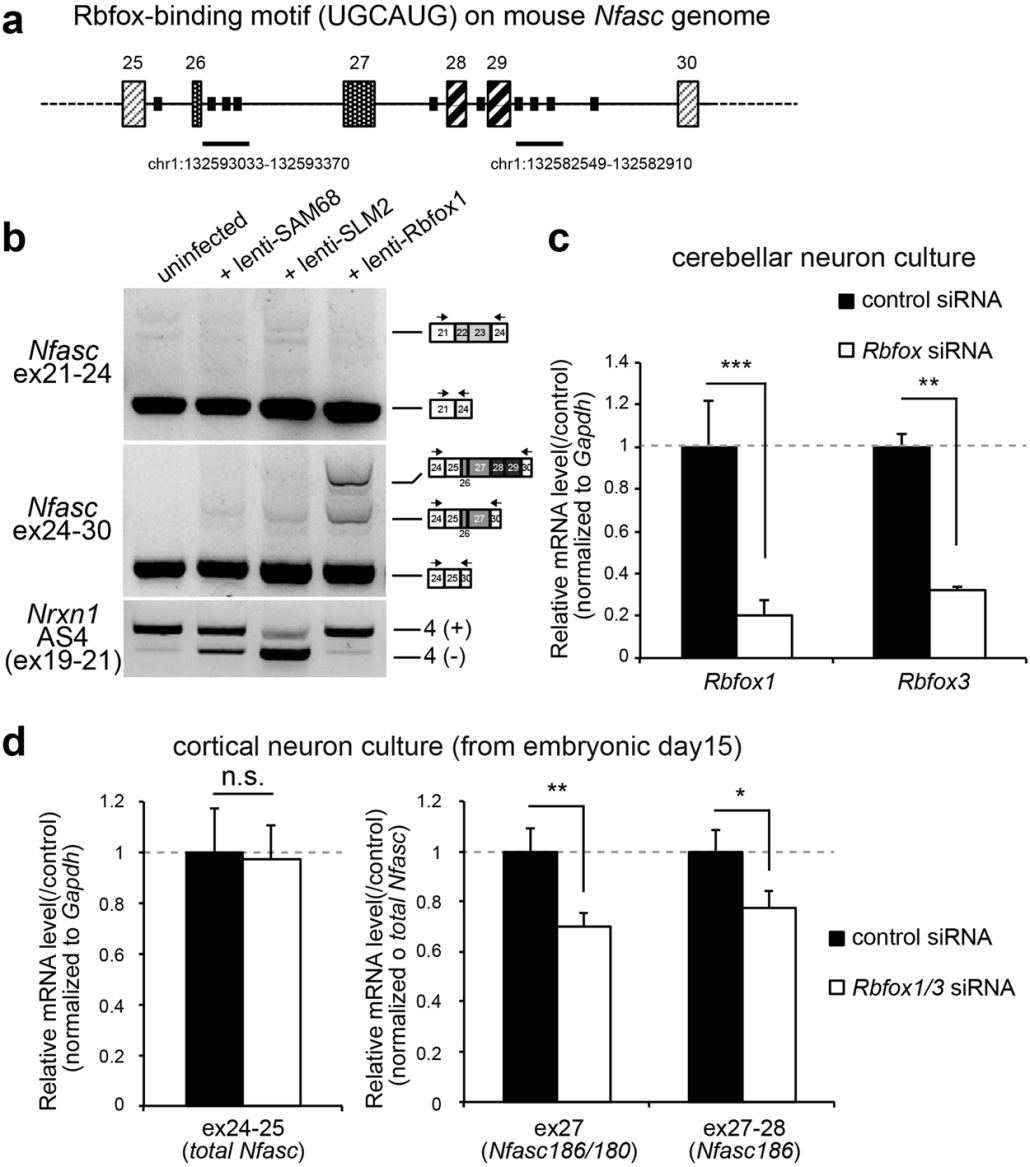
The Rbfox family induces inclusion of ex26-29 in neuronal *Nfasc*. (**a**) Position of the Rbfox-binding element UGCAUG on the mouse *Nfasc* genomic sequence on chromosome 1 (qE4). Underlines show the intronic sequence, which had been isolated using a previously reported CLIP assay^15^. (**b**) Shift in alternative splicing at *Nfasc* ex26-29 in cerebellar GCs overexpressing Rbfox1. Neuronal splicing factors, SAM68, two SAM-like molecules, SLM1 and SLM2, and Rbfox1 were overexpressed in cerebellar GC cultures via lentiviral infection. Rbfox1 expression induced inclusion of *Nfasc* ex26-29 both selectively and markedly. In contrast, neither SAM68 nor SLMs had any effect on *Nfasc* splicing. Inclusion of exon20 at *Nrxn1* at AS4 was accessed to confirm selective splicing activity of SAM68 and SLM proteins. (**c**) *Rbfox1* and *Rbfox3* knockdowns in cerebellar GC cultures by cell permeable siRNAs (1 μM, respectively). RT-qPCR confirmed that endogenous transcripts of *Rbfox1* and *Rbfox3* were reduced by approximately 80%. (*n* = 4 cultures). Values for the controls were set to 1.0. (**d**) Effect of double-knockdown by *Rbfox1/3* siRNAs on splicing of *Nfasc* in cultured cortical neurons. A qRT-PCR study showed that double-knockdown of *Rbfox1* and *Rbfox3* significantly attenuated the inclusion of ex27-28 without affecting the total transcript level of *Nfasc* (ex24-25). (*n* = 4 cultures). Values for the controls were set to 1.0.

We next sought to determine the factor responsible for the spatio-temporal manner of *Nfasc* splicing. We noticed that the intronic sequence neighbouring ex26-29 contained multiple copies of the Rbfox-binding consensus sequence, UGCAUG (Fig. 6a). In fact, a recent study that employed high-throughput sequencing together with UV crosslinking and immunoprecipitation (CLIP) listed clustered sequences downstream of exons 26 and 29 as putative Rbfox-interacting elements^15^ (Fig. 6a, underlines). The effect of the Rbfox family on splicing activation or repression depends on the location and context of the interacting sequences; downstream intronic binding sites are usually enhancers^16^. Therefore, we decided to test whether the Rbfox family induced the inclusion of ex26-29 in *Nfasc*. Interestingly, and similar to the effect of depolarization on *Nfasc* splicing, overexpression of Rbfox1 by lentiviral infection in cerebellar GC cultures strongly induced the insertion of ex26-29 in endogenous *Nfasc* transcripts (Fig. 6b). Moreover, neither the overexpression of the other splicing factors, SAM68 or SLMs, affected the splicing of *Nfasc*. To verify the involvement of the Rbfox family in *Nfasc* splicing further, we then tested the effect of *Rbfox1* and *Rbfox3* knockdowns in cultured cortical neurons, where NF186 is abundantly expressed. We initially confirmed that cell-permeable siRNA successfully reduced *Rbfox1* (approx. 80% reduction) and *Rbfox3* (approx. 70% reduction) transcripts in cerebellar GC culture (Fig. 6c). We found that an *Rbfox1/3* double-knockdown reduced inclusion of ex26-29 in *Nfasc* transcripts in cortical neuron cultures, without affecting total *Nfasc* levels (Figs 6d, S2), although knockdown of *Rbfox1* alone did not significantly reduce the levels (data not shown), suggesting that the Rbfox family synergistically contributes to the basal expression of *Nfasc186*.

**Figure 7:**
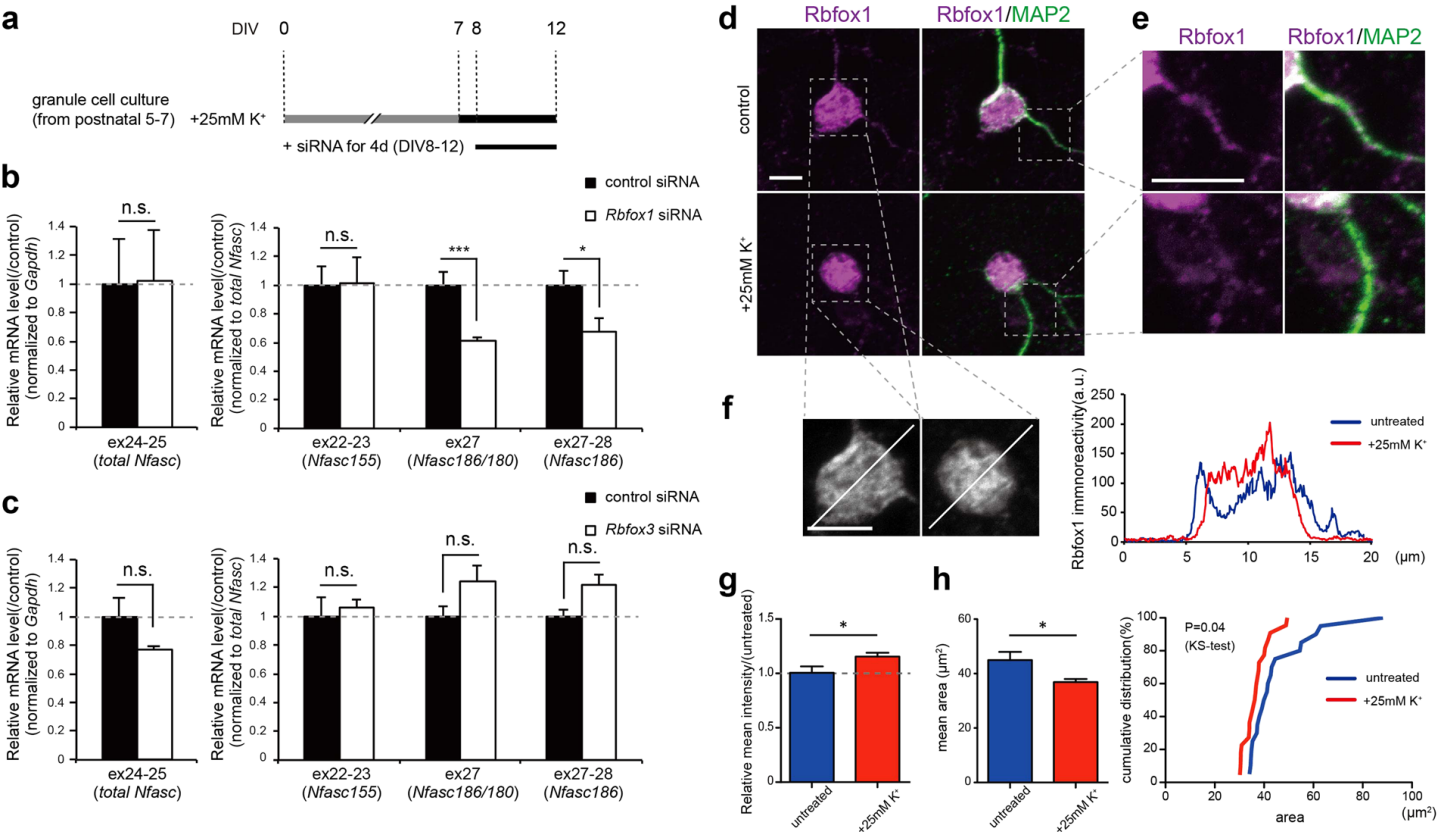
Rbfox1 selectively induces depolarization-dependent inclusion of ex26-29 in neuronal *Nfasc*. (**a**) Schematic diagram of the knockdown experiment by cell permeable *Rbfox1* siRNA in a high K^+^-treated neuronal culture. Cerebellar GCs were maintained in high-K^+^ (30 mM) media from 7 DIV through 12 DIV to induce inclusion of ex26-29. Rbfox and control siRNAs were applied for the last 4 days before harvest. (**b,c**) Knockdown effect of *Rbfox1* and *Rbfox3* siRNAs on alternative splicing of Nfasc. (**b**) Knockdown of *Rbfox1* significantly attenuated the high K^+^-induced inclusion of ex27-28 without affecting the total *Nfasc* transcript (ex24-25) levels. (**c**) In contrast, knockdown of *Rbfox3* did not have an effect on the inclusion. (n = 4 cultures). Values for the controls were set to 1.0. (**d–f**) Representative images of subcellular localization of Rbfox1 protein in untreated and depolarized cerebellar GCs. (**d**) Cultures were stained with anti-Rbfox1 antibody 1D10. MAP2 was co-stained to visualize overall morphology of the cultured neurons. Scale bar = 5 µm. (**e**) High magnification images of dendritic processes. Scale bar = 5 µm. (**f**) High magnification images of cell somas and the line scans from untreated and depolarized cerebellar GCs displaying immunoreactivity for Rbfox1 on these cell somas. Scale bar = 5 µm. (**g**) Relative mean intensity of Rbfox1 immunoreactivity on cell somas in untreated and depolarized cerebellar GCs. (n > 20 neurons each group in two sister cultures). Values for untreated cultures were set to 1.0. (**h**) Rbfox1-localized area (left) and the cumulative probability distribution (right) on the cell somas in control and depolarized cerebellar GCs. (n > 20 neurons each group in two sister cultures).

Importantly, it has also been reported that Rbfox1 regulates the depolarization-dependent alternative splicing of NMDA receptor 1^17^. Therefore, we next tested whether the Rbfox family was involved in the depolarization-dependent alternative splicing of *Nfasc*, as shown in Fig. 7a. Knockdown of Rbfox1 significantly reduced the high K^+^-induced inclusion of ex26-27 and ex28-29 in *Nfasc* transcripts (Figs 7b, S2). In contrast, *Nfasc* ex22-23, which is uniquely included in the glial isoform, NF155, was not influenced by knockdown of *Rbfox1*. We also found that, unlike *Rbfox1*, *Rbfox3* knockdown did not have an effect on the depolarization-dependent alternative splicing of *Nfasc* (Figs 7c, S2). Given our observation that *Nfasc* splicing is regulated through CaMK and ERK/MAPK signalling (Fig. 5e), these data suggest that Rbfox1 may selectively regulate the depolarization-dependent alternative splicing of *Nfasc* ex26-29 through a combination of CaMK and ERK/MAPK signalling.

Finally, to explore the cellular mechanisms underlying the depolarization-induced switch of *Nfasc* splicing by Rbfox1 protein, we examined the expression level and subcellular localization of Rbfox1 protein in cerebellar GCs upon depolarization. Western blot analysis showed no significant difference in Rbfox1 protein levels between untreated and high-K^+^-treated cultures (Fig. S3), but immunostaining with anti-Rbfox1 antibody in cerebellar GC culture revealed distinct staining patterns between untreated GCs and high-K^+^-treated GCs (Fig. 7d–f). Rbfox1 proteins were widely distributed throughout the neurons in untreated cultures (Fig. 7d), with immunoreactivity clearly detected on small dendritic processes of GCs (Fig. 7e). On the other hand, in high-K^+^-treated GCs, immunoreactivity for Rbfox1 proteins were not as clearly localised (Fig. 7e), but was more evident (Fig. 7f, g) and strongly concentrated (Fig. 7h) in the cell soma/nucleus, despite the absence of significant morphological changes in the somata (Fig. S4). Thus, depolarization induced-inclusion of *Nfasc* ex26-29 could be followed by dynamic changes in subcellular localization of Rbfox1 proteins. In addition to the knockdown studies, these data further suggest the role of Rbfox1 in dynamic regulation of *Nfasc* alternative splicing in mature cerebellar neurons.

## Discussion

This study systematically characterized the detailed alternative splicing patterns of *Nfasc* and its regulation in adult brains. First, we showed that alternative splicing of neural *Nfasc* was not only temporally dependent, as previously shown, but that it was also spatially dependent, showing a distinct pattern in the adult CNS (Figs 1 and 2). In particular, we showed that the alternative splicing patterns of neural *Nfasc* were cell type-specific in the cerebellum (Fig. 4), and that these patterns could be altered by high K^+^-induced depolarization (Fig. 5). Finally, an RNA-binding protein, Rbfox1, was shown to be responsible for the alternative splicing of neural *Nfasc* at ex26-29 (Figs 6 and 7). Together, this study revealed that alternative splicing of *Nfasc* in the mouse brain is more complex and dynamic than previously thought.

To date, it had been shown that the ratio of neural NF isoform expression changes during development, with the majority of NF140 (NF166) and NF180 switching to NF186^7, 8^. Our study revealed that this is not exactly the case in some brain parts. We unexpectedly found that a significant amount of the embryonic isoform, NF140, continued to be expressed in the caudal parts of the adult brain, where *Nfasc186* levels were reciprocally lower (Figs 1 and 2). Importantly, we showed that inclusion of ex26-29 was not comprehensive, in particular in cerebellar GCs, during development (Fig. 3). We speculate that the distinct expression pattern of *Nfasc* isoforms observed in different neuron types in adults might be due to the extent of the developmental switch in *Nfasc* splicing.

The molecular mechanism underlying *Nfasc* splicing is beginning to be uncovered. Indeed, a recent study revealed that Quaking (QKI) RNA-binding proteins are crucial regulators of the alternative splicing of glial *Nfasc* at ex21-22, demonstrating a role for QKI in oligodendrocyte development and myelination through *Nfasc* splicing^18^. Importantly, this study further identified Rbfox as a potential key regulator for neural *Nfasc*. Rbfox-family proteins (Rbfox1/2/3) are evolutionarily conserved regulators of tissue-specific alternative splicing^19^. We found that inclusion of ex26-29 was significantly attenuated by double-knockdown of *Rbfox1* and *Rbfox3* in cortical neuron cultures, where a large amount of *Nfasc186* is constitutively expressed (Fig. 6d). In support of our findings, CLIP-tagged Rbfox-binding consensus elements are observed on intronic sequences neighbouring ex26-29^15^. A recent report revealed that Rbfox regulates an alternative splicing program underlying the developmental switch associated with neural differentiation in the developing cortex^20^. Given that *Rbfox1* expression is upregulated during development^21^, the Rbfox family can potentially regulate the developmental switch in *Nfasc* splicing.

Expression of the Rbfox family overlaps widely, but is distinct in specific types of neurons, such as hippocampal interneurons and cerebellar neurons^22, 23^. In the cerebellum, whereas GCs express Rbfox1/3, Purkinje cells express Rbfox1/2. Therefore, the distinct levels of *Nfasc186* between GCs and Purkinje cells shown in Fig. 4 might be due to the prominent expression or regulatory activity of Rbfox2 in Purkinje cells. *Rbfox1/2*-knockout (KO) mice exhibit a strong functional deficit of AIS in Purkinje cells, a molecular phenotype that is shared with *Nfasc*-KO mice, suggesting that alternative splicing by Rbfox is required for Purkinje cell pacemaking^24^. Gehman and colleagues suggested that the impairments associated with *Rbfox1/2*-KO were mainly caused by altered *Scn8a* splicing. Our findings further imply that altered *Nfasc* splicing may also be the cause of functional AIS deficits. Therefore, we speculate that alternative splicing patterns of *Nfasc* ex26-29 may be at least partially dependent on the spatio-temporal expression pattern and protein dose of Rbfox-family proteins.

Importantly, our current study further revealed that Rbfox1 selectively contributes to the depolarization-dependent alternative splicing of *Nfasc* (Fig. 7), since *Rbfox1* knockdown, but not *Rbfox3*, selectively and strongly influenced inclusion of *Nfasc* ex26-29 in GCs exposed to high K^+^. Importantly, we further found that subcellular localization of Rbfox1 protein in GCs was markedly altered upon depolarization (Fig. 7d−h); whereas distribution of Rbfox1 proteins were widely extended toward small dendritic processes in mature GCs, they were strongly concentrated in the cell soma upon depolarization. Therefore, we speculate that depolarization-dependent inclusion of ex26-29 is likely due to the increase in nuclear concentration of Rbfox1 proteins through changes in the subcellular localization. Similar to this study, it has previously been reported that Rbfox1 regulates depolarization-dependent NMDA-R splicing in P19 cells through subcellular localization changes^17^. It has been suggested that the skipping of 53 nucleotides of exon 18 (ex18) on *Rbfox1* pre-mRNA during depolarization enhances nuclear localization of Rbfox1, subsequently triggering Rbfox binding element-dependent alternative splicing. However, splicing of ex18 on *Rbfox1* was not significantly changed by depolarization in cerebellar GCs under our experimental conditions (data not shown). It has been revealed that depolarization-dependent NMDA-R splicing by Rbfox1 is mediated via CaMK activity^17^. On the other hand, we revealed that depolarization-dependent *Nfasc* splicing was mediated via the ERK/MAPK pathway predominantly, rather than via the CaMK pathway (Fig. 5e). Thus, although the exact mechanism underlying subcellular localization of Rbfox1 in cerebellar GCs remains uncertain, our findings imply the existence of a similar but somewhat distinct mechanism underlying depolarization-induced alternative splicing by Rbfox1.

It has previously been shown that NF140 (NF166) is an embryonic isoform in the developing brain^11^, and that it regulates neurite outgrowth in chicken DRG via interaction with axonin-1^25^. However, our finding that NF140 is highly expressed in some regions of the adult mouse brain raises intriguing questions about the possible function of an embryonic isoform in the mature CNS. NF140 lacks the FN5 and PAT domains included in NF186. A recent study showed that these domains are likely unnecessary for AIS targeting or for sodium channel clustering. Two neural isoforms expressed in *Nfasc*-KO background do not show distinct subcellular localizations and function at the AIS and nodes of Ranvier^11^. If this were the case, it would indicate that NF140 could play a role similar to NF186 in adults. However, unknown functional differences may exist between NF186 and NF140. One possibility is shaping the structural and functional specificity of the AIS at the cell-type level. Previous reports have shown that NF140 (NF166) preferentially forms and stabilizes assemblies of GABAergic synapses on the AIS through clustering of gephyrin and GABAα receptor^10, 26^. Given that the inclusion of ex26-29 is distinct at the cell-type level in the adult cerebellum, *Nfasc* splicing may specify the unique functional and structural features of axo-axonic GABAergic input onto the AIS that are particular to a given type of neuron.

Another related question concerns the potential roles of activity-dependent shifts in NF isoforms. This study showed that alternative splicing of neural *Nfasc* is altered by high K^+^-induced depolarization in cerebellar GCs (Fig. 5). Depolarization-dependent alternative splicing may serve as a programmed mechanism for the dynamic modification of neuronal function, where the production of specific splice variants modifies cellular trafficking, signalling properties, or synaptic protein function^6^. Importantly, the resting membrane potential of immature GCs developmentally decreases from −25 mV to −55 mV upon maturation^27^. Membrane potential-dependent changes in intracellular Ca^2+^ control the gene expression profile in GCs^28^. Our previous study further revealed that depolarization controls the spatial and developmental regulation of neurexin-1 (*Nrxn1*) alternative splicing via SAM68 activation in cerebellar GCs, and that the changes in *Nrxn* splicing parallel the developmental shift in resting membrane potential in cerebellar GCs^14^. However, unlike *Nrxn* splicing, our current findings revealed that *Nfasc* splicing was not developmentally shifted in GCs (Fig. 4b). This difference might be explained by a distinct temporal expression of SAM68 and Rbfox1 in cerebellar GCs; whereas SAM68 is expressed robustly throughout development^14^, Rbfox1 is mainly expressed in mature internal GCs^24^. Therefore, depolarization-dependent alternative splicing by Rbfox1 is likely a mechanism mediating homeostasis in mature neurons, rather than inducing a developmental switch. Chronic depolarization dynamically changes the mobility and morphology of the AIS, which is thought to be a homeostatic mechanism for tuning the level of neuronal activity^29, 30^. Considering that NF is one of the key players in AIS structure and function, we speculate that a depolarization-induced NF isoform shift is a core mechanism underlying activity-dependent changes in the mobility and morphology of the AIS.

## Materials and Methods

### Antibodies and DNA constructs

For immunoblot analysis, the following commercially available antibodies were used: rabbit anti-neurofascin (ab31457, Abcam, Cambridge, MS, USA), rabbit anti-GAPDH (G9545, Sigma-Aldrich, St Louis, MO, USA), rabbit anti–SAM68^31^, rabbit anti-MAP2 (M3696, Sigma-Aldrich), and mouse anti-Rbfox1 (1D10, Millipore, Billerica, MS, USA). pEGFP-HA-neurofascin186 (Plasmid #31061)^32^ was purchased from Addgene (Cambridge, MA, USA). pEGFP-HA-neurofascin140 was constructed from this by removing the ex26-29 sequence.

### Lentivirus production

Vesicular stomatitis virus glycoprotein (VSV-G) pseudotyped lentiviral vectors provided by St. Jude’s Children’s Research Hospital^33^ were used in this study. The pCL20c vectors were designed under the control of the murine stem cell virus (MSCV) promoter. The viral vector was produced by co-transfection of human embryonic kidney cells (HEK293T) with a mixture of four plasmids using a calcium phosphate precipitation method. The four-plasmid mixture consisted of 6 µg of pCAG-kGP1R, 2 µg of pCAG-4RTR2, 2 µg of pCAG-VSV-G, and 10 µg of vector plasmid pCL20c MSCV-SLM1-2A-venus. The medium containing vector particles was harvested 40 h after transfection. Medium samples were concentrated by centrifugation at 25,800 rpm for 90 min. Virus samples were then suspended in cold phosphate-buffered saline (pH 7.4), frozen in aliquots, and stored at −80 °C until use. After assessing the titre in HEK293T cells, the appropriate amount of lentivirus was infected into cultured neurons 5 days before harvesting.

### RNA isolation and alternative-splicing assays

RNA was isolated with RNAiso Plus reagent (TaKaRa, Tokyo, Japan), followed by removal of contaminating DNA using Turbo DNA-free (RNase-free DNase, Ambion). Two micrograms of total RNA was reverse transcribed using random hexamers and ImProm-II (Promega, Madison, WI, USA). For semi-quantitative PCR, DNA fragment intensities were quantified using an image analyser (FAS-III, Toyobo, Osaka, Japan) and ImageGauge software (Fujifilm, Valhalla, NY, USA). The value for the intensity of each *Nfasc* isoform band was normalized to that of *total Nfasc* (ex24-25). Quantitative PCR (qPCR) was performed on a StepOnePlus qPCR system (Applied Biosystems, Waltham, MS, USA) with Power SYBR Green PCR Master Mix (Applied Biosystems) and the comparative C^T^ method. For the relative quantification by qRT-PCR, transcript level was normalized to that of *Gapdh*. On the other hand, transcript levels of each *Nfasc* isoform was normalized to that of *total Nfasc* (ex24-25), to avoid confounding by differences in amount of total *Nfasc* between groups. All the oligonucleotide primer sequences used for semi-quantitative PCR and qRT-PCR are shown in Table 1. Primers for Nrxn1/2/3 have been previously described^14^.

### Neuronal cell culture

Cerebellar GC culture were prepared from both male and female ICR mouse pups on postnatal days 5–7 by tissue dissociation with 0.05% trypsin (Sigma-Aldrich) in the presence of DNase I (Roche Applied Science, Penzberg, Germany) for 10 min at 37 °C. After cell dissociation, trypsin was inactivated with soybean trypsin inhibitor (Sigma−Aldrich). Cells were then plated into poly-D-lysine–coated dishes (0.5–1.0 × 10^5^/cm^2^) and maintained for 15 days in Neurobasal Medium (Invitrogen, Waltham, MA, USA) containing 2% B27 supplement, 2 mM Glutamax, and penicillin/streptomycin (Invitrogen). For pharmacological experiments, KN93 and U0126 were purchased from TOCRIS (Bristol, UK). For knockdown experiments, the following commercially available cell-permeable siRNAs were used: Accell non-targeting control siRNA (individual, D-001910-01, GE Healthcare Dharmacon, Lafayette, CO, USA) Accell mouse Rbfox1 siRNA (individual, A-041929-13, GE Healthcare Dharmacon) and Accell mouse Rbfox3 siRNA (individual, A-065754-09, GE Healthcare Dharmacon). Cultured cerebellar neurons were fixed with 4% paraformaldehyde in phosphate-buffered saline (PBS) for 20 min. After fixation, neurons were then permeabilized with the PBS containing 0.15% TritonX-100 for 15 min at room temperature and incubated with blocking solution (5% normal goat serum in PBS) for at least 30 min at room temperature and were then incubated with the primary antibodies for 24 h at 4 °C. For visualization, appropriate secondary antibodies conjugated to Alexa 546 or 488 (goat, 1:1000) (Life Technology) were used. Confocal images were captured on a LSM700 confocal system (Zeiss, Oberkochen, Germany). The original images were analysed using ImageJ software (NIH, Bethesda, MD, USA). After the appropriate threshold was set, the mean intensity and positive areas of immunoreactivity was measured on cell bodies of cerebellar GCs. Neuron morphology was visualized by staining for a neuronal marker, microtubule-associated protein 2 (MAP2).

All procedures related to the care and treatment of animals were carried out strictly in accordance with the Guide for the Care and Use of Laboratory Animals of Tokai University. All mice were maintained under specific pathogen-free conditions at the Laboratory Animal Center, Tokai University. The experimental protocol was approved by the Institutional Animal Care and Use Committee of Tokai University (permit number 141018). Surgeries were performed under sodium pentobarbital anaesthesia, and all efforts were made to minimize animal suffering.

### Biochemical analysis

Cells or brain tissues were lysed with RIPA buffer (25 mM Tris-HCl, pH 8.0, 150 mM NaCl, 1% NP-40, 1% deoxycholate, 0.1% SDS) containing a protease inhibitor cocktail (Roche Applied Science). For protein interaction studies, soluble fractions were subjected to immunoprecipitation for 24 h at 4 °C and analysed by immunoblotting. For visualisation, a horseradish peroxidase-conjugated secondary antibody and enhanced chemiluminescence detection (Pierce, Dallas, TX, USA) were used, with signals acquired via an image analyser (LAS500; GE Healthcare).

### Laser-capture microdissection

Brains were obtained from adult mice (2–3-months-old) and freshly frozen in powdered dry ice. Frozen sections cut at a thickness of 10 µm on a cryostat (CM1900; Leica) were mounted on silane-coated glass slides (Muto Pure Chemicals, Tokyo, Japan) and air-dried. Cresyl violet staining was then performed to visualize the neuronal structure in brain. Approximately 500−800 µm^2^ of area from each layer per animal was sculpted with a laser microdissection system (Carl Zeiss MicroImaging, Jena, Germany) within 2 h. Thereafter, total RNA was extracted from the dissected specimen using a NucleoSpin RNA XS Kit (TaKaRa).

### Statistical analysis

GraphPad Prism 5 (GraphPad Software, Inc., La Jolla, CA, USA) was used for most of the statistical analyses. Pairwise comparisons were performed using a Student’s *t*-test. For multiple comparisons, an analysis of variance (ANOVA) followed by Bonferroni’s or Dunnet’s tests was used. Data are represented as the mean ± standard error of the mean. Significance is indicated as follows: ***p < 0.001; **p < 0.01; *p < 0.05.

## Electronic supplementary material

Supplementary Info
