# Supplementary material for: Spatio-temporal and dynamic regulation of neurofascin alternative splicing in mouse cerebellar neurons
Source: Sci Rep. 2017 Sep 12;7:11405. doi: 10.1038/s41598-017-11319-5 (PMC5595909; doi:10.1038/s41598-017-11319-5)
Supplement: Supplementary file 1 — Supplementary Info [file 41598_2017_11319_MOESM1_ESM.pdf]

Supplemental Information

Spatio-temporal and dynamic regulation of neurofascin alternative splicing in mouse cerebellar neurons

Satoko Suzuki, Noriko Ayukawa, Chisa Okada, Masami Tanaka, Susumu Takekoshi, Yoko Iijima, and Takatoshi Iijima

Supplemental Figure legends

Figure S1. Inclusion of *Nfasc* ex26-29 caused by high K^+-induced depolarization

Cultured GCs were treated with addition of 25 mM K^+ (final concentration: 30 mM) for 1 h, 3 h, 6 h, 1 day, 3 days or 5 days. All the cultures were harvested at DIV12. Brain-derived neurotrophic factor (*Bdnf*) expression was monitored to confirm immediate-early gene induction.

Figure S2. Representative gel images of the knockdown effect of *Rbfox1* and *Rbfox3* siRNAs on alternative splicing of *Nfasc* (related to Figs. 6d, 7b, and 7c).

PCR products from the same samples used for qRT-PCR (Figs. 6d, 7b, and 7c) were loaded onto agarose gels to confirm the knockdown effects.

Figure S3. Western blot analysis of total cell lysates from untreated control and depolarized GC cultures with anti-Rbfox1 antibody.

Protein levels of Rbfox1 were normalized to that of ubiquitously expressed SAM68. (n = 3 cultures)

Figure S4. Areas of somata (left) and nuclei in control and depolarized cerebellar GCs (related to Fig. 7h).

(a) The mean area and cumulative distribution of $MAP2^+$ somata. There was no significant difference in size of $MAP2^+$ somata (n >20 neurons each group in two sister cultures)

Figure S5. Uncropped images of blots presented in Figures 1−7.

(a1)–(a3) Fig 1b. (b1)–(b5) Fig 2b. (c1)–(c4) Fig 3a. (d1)–(d3) Fig 4a. (e) Fig 4b. (f1)–(f2) Fig 4d. (g1)–(g5) Fig 5b. (h1)–(h2) Fig 5d. (i1)–(i3) Fig 5e. (j1)–(j2) Fig 6b. Images show agarose gels for splicing assays and the membranes used in western blotting with the indicated antibodies.

Figure S6. Uncropped images of blots presented in Figures S1-4.

(a1)–(a5) Fig S1. (b1)–(b9) Fig S2. (c1)–(c2) Fig S3. Images show agarose gels for

splicing assays and the membranes used in western blotting with the indicated antibodies.

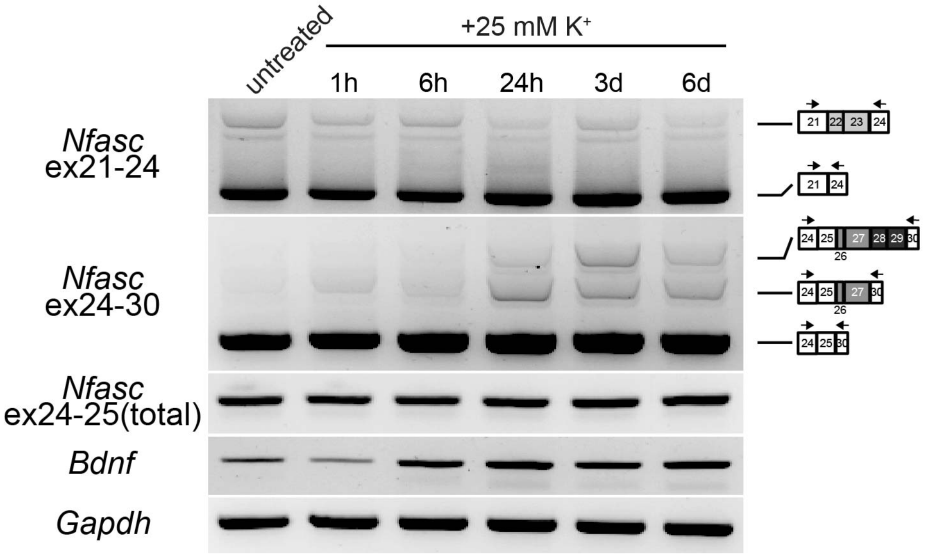
untreated
+25 mM K+
1h
6h
24h
3d
6d
Nfasc ex21-24
Nfasc ex24-30
Nfasc ex24-25(total)
Bdnf
Gapdh
21 22 23 24
21 24
24 25 27 28 29 30
26
24 25 27 30
26
24 25 30

Figure S1

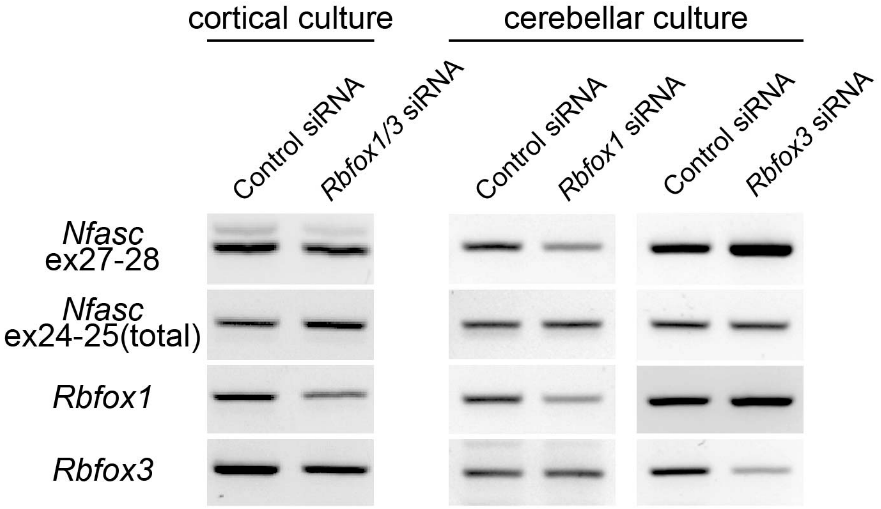
cortical culture
cerebellar culture
Control siRNA
Rbfox1/3 siRNA
Control siRNA
Rbfox1 siRNA
Control siRNA
Rbfox3 siRNA
Nfasc ex27-28
Nfasc ex24-25(total)
Rbfox1
Rbfox3

Figure S2

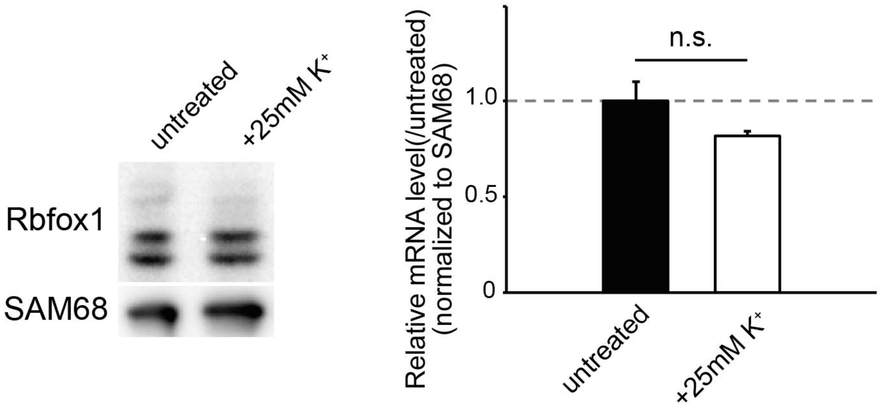
untreated
+25mM K+
Rbfox1
SAM68
Relative mRNA level(/untreated)
(normalized to SAM68)
n.s.
1.0
0.5
0
untreated
+25mM K+

Figure S3

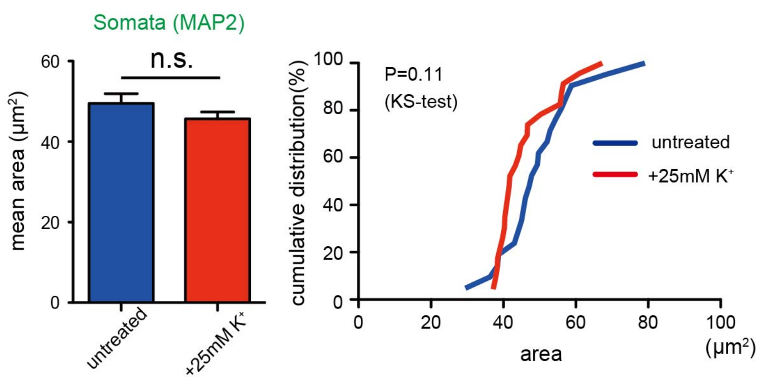
Somata (MAP2)
n.s.
mean area (μm²)
60
40
20
0
untreated
+25mM K⁺
P=0.11
(KS-test)
cumulative distribution(%)
100
80
60
40
20
0
0
20
40
60
80
100
area
(μm²)
untreated
+25mM K⁺

Figure S4

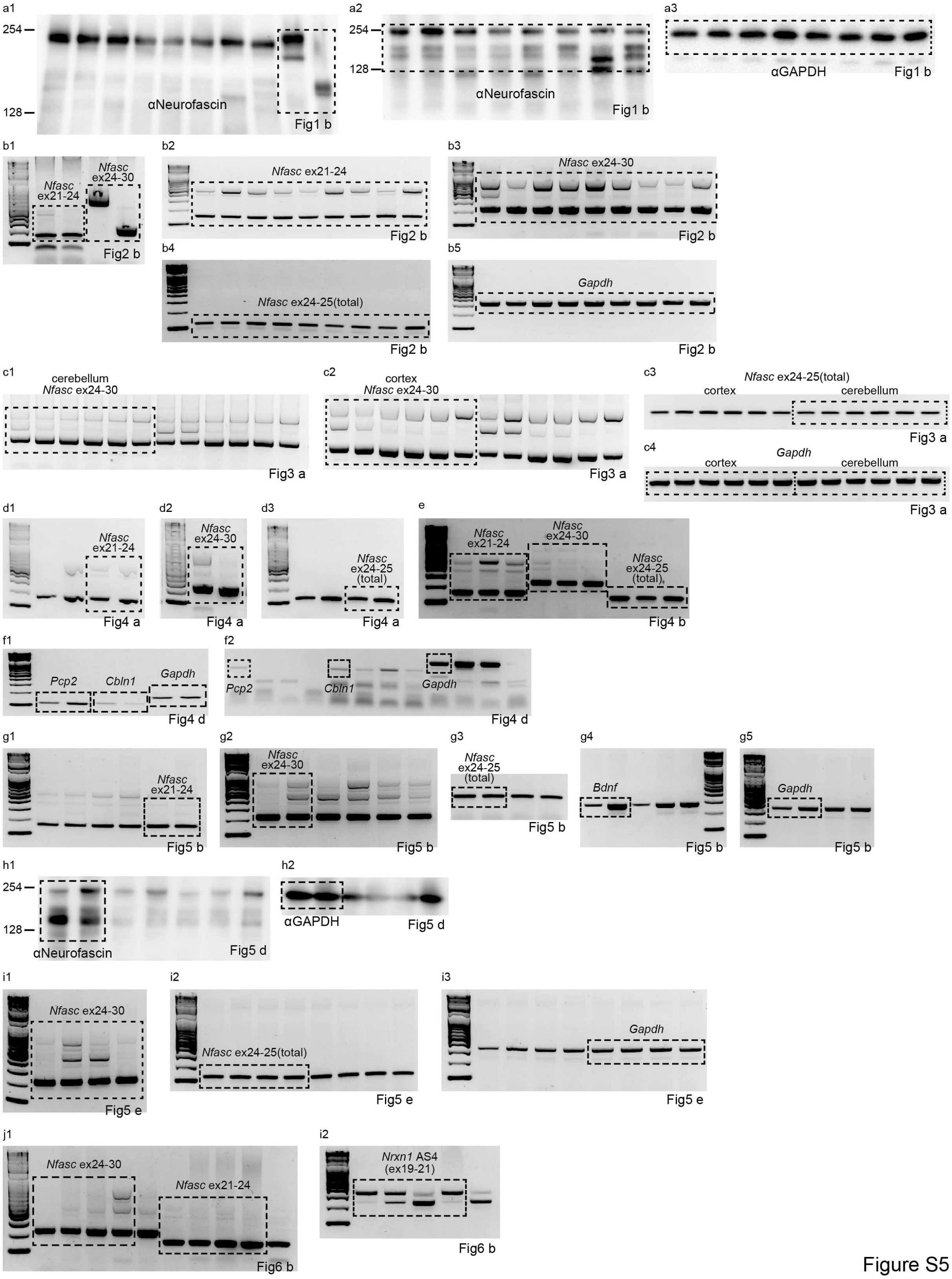
a1
254
128
αNeurofascin
Fig1 b
a2
254
128
αNeurofascin
Fig1 b
a3
αGAPDH
Fig1 b
b1
Nfasc ex21-24
Nfasc ex24-30
Fig2 b
b2
Nfasc ex21-24
Fig2 b
b3
Nfasc ex24-30
Fig2 b
b4
Nfasc ex24-25(total)
Fig2 b
b5
Gapdh
Fig2 b
c1
cerebellum
Nfasc ex24-30
Fig3 a
c2
cortex
Nfasc ex24-30
Fig3 a
c3
Nfasc ex24-25(total)
cortex
cerebellum
Fig3 a
c4
cortex
Gapdh
cerebellum
Fig3 a
d1
Nfasc ex21-24
Fig4 a
d2
Nfasc ex24-30
Fig4 a
d3
Nfasc ex24-25 (total)
Fig4 a
e
Nfasc ex21-24
Nfasc ex24-30
Nfasc ex24-25 (total)
Fig4 b
f1
Pcp2
Cbln1
Gapdh
Fig4 d
f2
Pcp2
Cbln1
Gapdh
Fig4 d
g1
Nfasc ex21-24
Fig5 b
g2
Nfasc ex24-30
Fig5 b
g3
Nfasc ex24-25 (total)
Fig5 b
g4
Bdnf
Fig5 b
g5
Gapdh
Fig5 b
h1
254
128
αNeurofascin
Fig5 d
h2
αGAPDH
Fig5 d
i1
Nfasc ex24-30
Fig5 e
i2
Nfasc ex24-25(total)
Fig5 e
i3
Gapdh
Fig5 e
j1
Nfasc ex24-30
Nfasc ex21-24
Fig6 b
i2
Nrxn1 AS4 (ex19-21)
Fig6 b

Figure S5

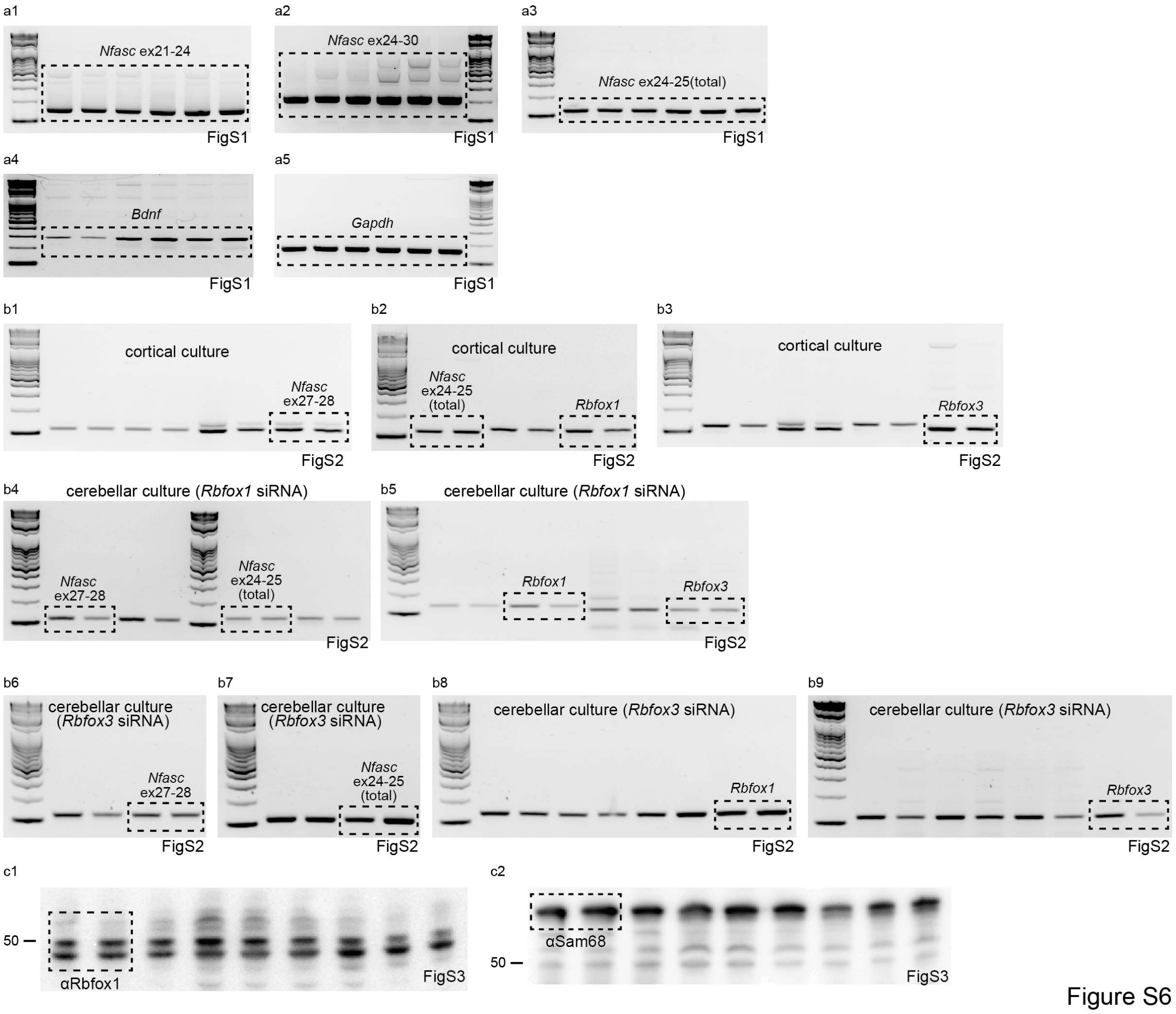
a1
Nfasc ex21-24
FigS1
a2
Nfasc ex24-30
FigS1
a3
Nfasc ex24-25(total)
FigS1
a4
Bdnf
FigS1
a5
Gapdh
FigS1
b1
cortical culture
Nfasc ex27-28
FigS2
b2
cortical culture
Nfasc ex24-25 (total)
Rbfox1
FigS2
b3
cortical culture
Rbfox3
FigS2
b4
cerebellar culture (Rbfox1 siRNA)
Nfasc ex27-28
Nfasc ex24-25 (total)
FigS2
b5
cerebellar culture (Rbfox1 siRNA)
Rbfox1
Rbfox3
FigS2
b6
cerebellar culture (Rbfox3 siRNA)
Nfasc ex27-28
FigS2
b7
cerebellar culture (Rbfox3 siRNA)
Nfasc ex24-25 (total)
FigS2
b8
cerebellar culture (Rbfox3 siRNA)
Rbfox1
FigS2
b9
cerebellar culture (Rbfox3 siRNA)
Rbfox3
FigS2
c1
50
αRbfox1
FigS3
c2
αSam68
50
FigS3

Figure S6
